# Supplementary material for: Posterior-based proposals for speeding up Markov chain Monte Carlo
Source: R Soc Open Sci. 2019 Nov 20;6(11):190619. doi: 10.1098/rsos.190619 (PMC6894579; doi:10.1098/rsos.190619)
Supplement: Appendices [file rsos190619supp1.pdf]

Appendices for "Posterior-based proposals for speeding up Markov chain Monte Carlo"

C. M. Pooley[1,2], S. C. Bishop[3], A. Doeschl-Wilson[1] and G. Marion[2]

1 The Roslin Institute, The University of Edinburgh, Midlothian, EH25 9RG, UK.

2 Biomathematics and Statistics Scotland, James Clerk Maxwell Building, The King's Buildings, Peter Guthrie Tait Road, Edinburgh, EH9 3FD, UK.

3 Deceased.

Appendix A: Importance sampling

Importance sampling (IS) is a general technique for estimating properties of a given distribution (which can't directly be sampled from) using samples generated from a different (more accessible) distribution [1, 2]. We emphasise that PBPs are *not* a type of IS, but do make use of importance distributions (IDs). Therefore a brief description is now given. The posterior in Eq.(1.1) can be expressed as

$$\pi(\theta,\xi \mid y)=\pi(\xi \mid \theta,y)\pi(\theta \mid y), \tag{A1}$$

which, using Eq.(2.1), may be written

$$\pi(\theta,\xi \mid y)=\pi(\theta \mid y)\prod_{e=1}^{E}\pi(\xi_e \mid \xi_{e'<e},\theta,y). \tag{A2}$$

This implies that, in principle at least, posterior samples can be generated by first sampling θ from $\pi(\theta|y)$, then ξ_1 from $\pi(\xi_1|\theta,y)$, then ξ_2 from $\pi(\xi_2|\xi_1,\theta,y)$, and so on and so forth until a complete set of latent variables ξ is created. Unfortunately, however, these distributions are typically intractable, so cannot be directly sampled. To overcome this difficultly importance distributions (IDs) $f_{ID}(\theta|y)$ (which, for example, could be chosen to be multivariate normal) and $f_{ID}(\xi_e|\xi_{e'<e},\theta,y)$ (a set of univariate distributions, such as normal or Poisson, for each variable *e*) are defined which *can* be sampled from. IDs are chosen to resemble the true distributions as closely as possible.

IS consists of first sampling θ from $f_{ID}(\theta|y)$ and then successively drawing ξ_e from $f_{ID}(\xi_e|\xi_{e'<e},\theta,y)$ for *e*=1 to *E*. The resulting sample θ, ξ has an associated "weight"

$$w=\frac{\pi(y \mid \xi,\theta)\pi(\xi \mid \theta)\pi(\theta)}{f_{ID}(\theta \mid y)\prod_{e=1}^{E}f_{ID}(\xi_e \mid \xi_{e'<e},\theta,y)}, \tag{A3}$$

which accounts for the fact that it isn't a true posterior sample [1]. Repeating IS a sufficient number of times gives unbiased estimates for posterior quantities of interest. For example, if *i* indexes sample number then

$$\langle\theta\rangle=\sum_i \theta^i w^i \Big/ \sum_i w^i \tag{A4}$$

gives an estimate for parameter posterior means[1]. Unfortunately IS becomes highly inefficient for complex models because the vast majority of samples have negligible weight (leading to poor statistical estimates for quantities such as those in Eq.(A4)). This necessitates the use of MCMC approaches in the first place.

In summary, this appendix has identified importance distributions $f_{ID}(\xi_e|\xi_{e'<e},\theta,y)$ (which take standard functional forms such as normal, Poisson, *etc.*) that aim to account for both the model and observations by approximating $\pi(\xi_e|\xi_{e'<e},\theta,y)$. For example supposing that $\pi(\xi_e|\xi_{e'<e},\theta,y)$ has a normal-like distribution, the importance distribution would be chosen to be normal[2], *i.e.* $f_{ID}(\xi_e|\xi_{e'<e},\theta,y)= f_{\text{norm}}(\xi_e|\mu(\xi_{e'<e},\theta,y),\sigma(\xi_{e'<e},\theta,y))$ with mean μ and standard deviation σ functionally depend on $\xi_{e'<e}$, θ and y. Details on these functional dependencies are given in §4.

Appendix B: Derivation of PBPs for Poisson or normal IDs

Here we explicitly demonstrate the validity of the two conditions in Eq.(3.4) when using the sampling procedures outlined in Table 1 for the Poisson and normal IDs.

Poisson ID

For a model which utilises a Poisson ID for latent variable ξ_e, the following probability mass functions are defined:

$$\begin{aligned} f_{ID}(\xi_e^i|\xi_{e'<e}^i,\theta^i,y)&=\frac{\lambda_i^{\xi_e^i}e^{-\lambda_i}}{\xi_e^i!},\\ f_{ID}(\xi_e^p|\xi_{e'<e}^p,\theta^p,y)&=\frac{\lambda_p^{\xi_e^p}e^{-\lambda_p}}{\xi_e^p!},\end{aligned} \tag{B1}$$

where λ_i is some known function of $(\xi_{e'<e}^i,\theta^i,y)$ and λ_p is some known function of $(\xi_{e'<e}^p,\theta^p,y)$.

Suppose, arbitrarily, that the expected number of occurrences in the proposed state λ_p is greater than the expected number in the initial state λ_i. Table 1 shows that the number generated in the proposed state ξ_e^p is calculated using

$$\xi_e^p=\xi_e^i+X, \tag{B2}$$

where ξ_e^i is the number in the initial state and X is sampled from a Poisson distribution with average number given by the difference between λ_p and λ_i:

$$X\sim\text{Pois}\left(\lambda_p-\lambda_i\right). \tag{B3}$$

The probability of this proposal is given by

[1] The average of the weights themselves $\Sigma_i w^i/N$ gives an unbiased estimate of the model evidence $\pi(y)$.

[2] See appendix G for a definition of this distribution.

$$g(\xi_e^p) = \frac{\left(\lambda_p - \lambda_i\right)^{\xi_e^p - \xi_e^i} e^{-\left(\lambda_p - \lambda_i\right)}}{\left(\xi_e^p - \xi_e^i\right)!}. \tag{B4}$$

Interchanging *i* and *p* in Table 1 shows that the reverse transition is taken from a binomial probability distribution

$$\xi_e^i \sim B(\xi_e^p, \tfrac{\lambda_i}{\lambda_p}), \tag{B5}$$

with proposal probability

$$g(\xi_e^i) = \frac{\xi_e^p!}{\xi_e^i!(\xi_e^p - \xi_e^i)!}\left(\tfrac{\lambda_i}{\lambda_p}\right)^{\xi_e^i}\left(1 - \tfrac{\lambda_i}{\lambda_p}\right)^{\xi_e^p - \xi_e^i}. \tag{B6}$$

Combining the results from Eqs.(B4) and (B6) gives

$$\begin{aligned} \frac{g(\xi_e^i)}{g(\xi_e^p)} &= \frac{\xi_e^p!}{\xi_e^i!(\xi_e^p - \xi_e^i)!}\left(\tfrac{\lambda_i}{\lambda_p}\right)^{\xi_e^i}\left(1 - \tfrac{\lambda_i}{\lambda_p}\right)^{\xi_e^p - \xi_e^i} \times \left(\frac{\left(\lambda_p - \lambda_i\right)^{\xi_e^p - \xi_e^i} e^{-\left(\lambda_p - \lambda_i\right)}}{\left(\xi_e^p - \xi_e^i\right)!}\right)^{-1} \\ &= \frac{\lambda_p^{\xi_e^i} e^{-\lambda_i}}{\xi_e^i!} \times \left(\frac{\lambda_p^{\xi_e^p} e^{-\lambda_p}}{\xi_e^p!}\right)^{-1}. \end{aligned} \tag{B7}$$

Using this, along with Eq.(B1), finally gives

$$\frac{f_{ID}(\xi_e^p \mid \xi_{e'<e}^p, \theta^p, y)}{f_{ID}(\xi_e^i \mid \xi_{e'<e}^i, \theta^i, y)} \frac{g\left(\xi_e^i\right)}{g\left(\xi_e^p\right)} = \frac{\lambda_p^{\xi_e^p} e^{-\lambda_p}}{\xi_e^p!}\left(\frac{\lambda_i^{\xi_e^i} e^{-\lambda_i}}{\xi_e^i!}\right)^{-1} \frac{\lambda_p^{\xi_e^i} e^{-\lambda_i}}{\xi_e^i!}\left(\frac{\lambda_p^{\xi_e^p} e^{-\lambda_p}}{\xi_e^p!}\right)^{-1} = 1, \tag{B8}$$

which shows that condition 1 in Eq.(3.4) is, indeed, satisfied.

Condition 2 is satisfied because *X*=0 when $\lambda_p=\lambda_i$ in Eq.(B3), and so by definition $\xi^p=\xi^i$ in Eq.(B2) (similarly $\xi^i=\xi^p$ in Eq.(B5)).

Normal ID

Here we consider the case of a normal ID for latent variable ξ_e :

$$\begin{aligned} f_{ID}(\xi_e^i \mid \xi_{e'<e}^i, \theta^i, y) &= \frac{1}{\sqrt{2\pi}\sigma_i} e^{-\frac{(\xi_e^i - \mu_i)^2}{2\sigma_i^2}}, \\ f_{ID}(\xi_e^p \mid \xi_{e'<e}^p, \theta^p, y) &= \frac{1}{\sqrt{2\pi}\sigma_p} e^{-\frac{(\xi_e^p - \mu_p)^2}{2\sigma_p^2}}. \end{aligned} \tag{B9}$$

Suppose, arbitrarily, that the standard deviation in the proposed state σ_p is smaller than in the initial state σ_i. Table 1 shows that the proposal for ξ_e^p is given by

$$\xi_e^p \sim N\left(\mu_p + \alpha \tfrac{\sigma_p^2}{\sigma_i^2}(\xi_e^i - \mu_i), \kappa \tfrac{\sigma_p^2}{\sigma_i^2}(\sigma_i^2 - \sigma_p^2)\right), \tag{B10}$$

where $\alpha^2 = \kappa + (1-\kappa)\frac{\sigma_i^2}{\sigma_p^2}$ and κ is a tuneable constant. The probability density function for generating this final state is given by

$$g(\xi_e^p) = \frac{1}{\sqrt{2\pi\kappa \frac{\sigma_p^2}{\sigma_i^2}(\sigma_i^2 - \sigma_p^2)}} e^{-\frac{\left(\xi_e^p - \left(\mu_p + \alpha \frac{\sigma_p^2}{\sigma_i^2}(\xi_e^i - \mu_i)\right)\right)^2}{2\kappa \frac{\sigma_p^2}{\sigma_i^2}(\sigma_i^2 - \sigma_p^2)}}. \tag{B11}$$

We now consider the reverse transition. Since *p* and *i* are now switched in Table 1, the corresponding proposal probability density function is given by

$$g(\xi_e^i) = \frac{1}{\sqrt{2\pi\kappa(\sigma_i^2 - \sigma_p^2)}} e^{-\frac{\left(\xi_e^i - \left(\mu_i + \alpha(\xi_e^p - \mu_p)\right)\right)^2}{2\kappa(\sigma_i^2 - \sigma_p^2)}}. \tag{B12}$$

The ratio between Eqs.(B12) and (B11) is given by

$$\begin{aligned}
\frac{g(\xi_e^i)}{g(\xi_e^p)} &= \sqrt{\frac{\sigma_p^2}{\sigma_i^2}} e^{-\frac{1}{2\kappa(\sigma_i^2-\sigma_p^2)}\left[\left((\xi_e^i-\mu_i)-\alpha(\xi_e^p-\mu_p)\right)^2 - \frac{\sigma_i^2}{\sigma_p^2}\left((\xi_e^p-\mu_p)-\alpha\frac{\sigma_p^2}{\sigma_i^2}(\xi_e^i-\mu_i)\right)^2\right]} \\
&= \sqrt{\frac{\sigma_p^2}{\sigma_i^2}} e^{-\frac{1}{2\kappa(\sigma_i^2-\sigma_p^2)}\left[(\xi_e^i-\mu_i)^2+\alpha^2(\xi_e^p-\mu_p)^2-2\alpha(\xi_e^i-\mu_i)(\xi_e^p-\mu_p)-\left(\frac{\sigma_i^2}{\sigma_p^2}(\xi_e^p-\mu_p)^2+\alpha^2\frac{\sigma_p^2}{\sigma_i^2}(\xi_e^i-\mu_i)^2-2\alpha(\xi_e^p-\mu_p)(\xi_e^i-\mu_i)\right)\right]} \\
&= \sqrt{\frac{\sigma_p^2}{\sigma_i^2}} e^{-\frac{1}{2\kappa(\sigma_i^2-\sigma_p^2)}\left[\left(1-\alpha^2\frac{\sigma_p^2}{\sigma_i^2}\right)(\xi_e^i-\mu_i)^2+\left(\alpha^2-\frac{\sigma_i^2}{\sigma_p^2}\right)(\xi_e^p-\mu_p)^2\right]} \\
&= \sqrt{\frac{\sigma_p^2}{\sigma_i^2}} e^{-\frac{\sigma_i^2-\alpha^2\sigma_p^2}{2\kappa(\sigma_i^2-\sigma_p^2)}\left[\frac{1}{\sigma_i^2}(\xi_e^i-\mu_i)^2-\frac{1}{\sigma_p^2}(\xi_e^p-\mu_p)^2\right]}.
\end{aligned}$$

(B13)

The definition $\alpha^2 = \kappa + (1-\kappa)\frac{\sigma_i^2}{\sigma_p^2}$ can be rearrange to $\kappa\left(\sigma_i^2 - \sigma_p^2\right) = \sigma_i^2 - \alpha^2\sigma_p^2$, leading to

$$\begin{aligned}
\frac{g(\xi_e^i)}{g(\xi_e^p)} &= \sqrt{\frac{\sigma_p^2}{\sigma_i^2}} e^{-\frac{\sigma_i^2-\alpha^2\sigma_p^2}{2\kappa(\sigma_i^2-\sigma_p^2)}\left[\frac{1}{\sigma_i^2}(\xi_e^i-\mu_i)^2-\frac{1}{\sigma_p^2}(\xi_e^p-\mu_p)^2\right]} \\
&= \frac{1}{\sqrt{2\pi\sigma_i^2}}\left(e^{-\frac{1}{2\sigma_i^2}(\xi_e^i-\mu_i)^2}\right)\left[\frac{1}{\sqrt{2\pi\sigma_p^2}}\left(e^{-\frac{1}{2\sigma_p^2}(\xi_e^p-\mu_p)^2}\right)\right]^{-1}.
\end{aligned} \tag{B14}$$

This, together with the expressions in Eq.(B9), shows that condition 1 in Eq.(3.4) is, indeed, satisfied. Furthermore, condition 2 is satisfied by noting that the variance in the proposal in Eq.(B10) goes to zero when $\sigma_p^2 = \sigma_i^2$.

The validity of all the sampling procedures in Table 1 can be verified by following essentially the same procedure as above to show that the two conditions in Eq.(3.4) are satisfied.

Appendix C: Adaptation period

Posterior-based proposals contain two quantities in Eq.(3.6) that need to be established: a numerical approximation to the covariance matrix of the posterior Σ^θ, and a jumping size *j*. Motivated by adaptive MCMC [3, 4], these are calculated during an "adaptation" period, which also acts as the required burn-in phase (*i.e*. this ensures that the first sample drawn after the adaptation period is representative of a random draw from the posterior). In this study $I^{ad}=10^4$ iterations are used for adaptation, and subsequently Σ^θ and *j* are fixed[3].

Covariance matrix Σ_θ: During the adaptation period the number of MCMC iterations i changes from 1 to I^{ad}. An approximation to the posterior covariance matrix Σ^θ is calculated every *100* iterations based on samples from *i/2* to *i*:

$$\Sigma^\theta_{mn} = \frac{1}{\frac{1}{2}i - 1}\sum_{i'=\frac{i}{2}}^{i}(\theta^{i'}_m - \overline{\theta_m})(\theta^{i'}_n - \overline{\theta_n}), \quad \overline{\theta_m} = \frac{1}{\frac{1}{2}i}\sum_{i'=\frac{i}{2}}^{i}\theta^{i'}_m. \tag{C1}$$

This is effectively equivalent to using a dynamically changing burn-in period set at half the current iteration number. As the adaptation period progresses the estimated covariance matrix Σ^θ becomes a better and better approximation, which helps to improve the efficiency of the algorithm. For the first 100 samples Σ^θ is set to a diagonal matrix with elements chosen to be sufficiently small to ensure a good initial acceptance rate.

Jumping size *j*: This determines the acceptance rate for PBPs in Eq.(3.7). If *j* is too large very few proposals are accepted, and if too small mixing is slow. A robust heuristic method for optimising *j* is as follows. Initially *j* is set to a small quantity. Each time a PBP is accepted, *j* is updated according to

$$j^{new} = j \times 1.02, \tag{C2}$$

and when rejected

$$j^{new} = j \times 0.99. \tag{C3}$$

These numerical factors are chosen for two reasons: Firstly, the two updates in Eqs.(C2) and (C3) balance each other out when acceptance occurs around 33% of the time (which from appendix H was found to be approximately optimal), leading to a steady state solution for *j*. Secondly, they are sufficiently close to 1 to prevent large fluctuations in *j*, but sufficiently far to allow for the steady state solution to be found during the adaptation period.

[3] This ensures that detailed balance is strictly enforced when MCMC samples are taken.

Appendix D: Derivation of acceptance probability

We derive the expression in Eq.(3.7). Based on Eq.(1.1), the MH acceptance probability is given by

$$P_{MH} = \min\left\{1, \frac{\pi(y|\xi^p,\theta^p)\pi(\xi^p|\theta^p)\pi(\theta^p)}{\pi(y|\xi^i,\theta^i)\pi(\xi^i|\theta^i)\pi(\theta^i)} \frac{g^{p\to i}}{g^{i\to p}}\right\}, \tag{D1}$$

where $g^{i\to p}$ represents the proposal probability density for generating θ^p and ξ^p given the current state θ^i and ξ^i, and $g^{p\to i}$ represents the corresponding quantity in the opposite direction[4]. Following steps 1 and 2 in the PBP algorithm from §3.4, the overall proposal probability can be expressed as

$$g^{i\to p} = f_{MVN}(\theta^p - \theta^i, j^2\Sigma^\theta)\prod_{e=1}^{E} g\left(\xi_e^p\right). \tag{D2}$$

Substituting this, along with the reverse transition, into Eq.(D1) (and noting that the MVN distributions are symmetric[5]), gives

$$P_{MH} = \min\left\{1, \frac{\pi(y|\xi^p,\theta^p)\pi(\xi^p|\theta^p)\pi(\theta^p)}{\pi(y|\xi^i,\theta^i)\pi(\xi^i|\theta^i)\pi(\theta^i)} \prod_{e=1}^{E} \frac{g\left(\xi_e^p\right)}{g\left(\xi_e^i\right)}\right\}. \tag{D3}$$

Substituting condition 1 from Eq.(3.4) into this expression leads to the final result in Eq.(3.7).

Appendix E: Further insights into PBPs

Here we provide some additional notes on the PBP algorithm in §3.4:

Step 1: Strong posterior correlations can exist not only between model parameters and latent variables (as demonstrated in Fig. 1(a)), but also between different model parameters themselves (*i.e.* after marginalisation over latent variables). Equation (3.6) helps to mitigate against these, helping to further facilitating mixing. Other possibilities for proposals in parameter space are discussed in appendix F (along with complications such as what to do when parameters are discrete rather than continuous), and sampling from MVNs using Cholesky decomposition [5] is described in appendix G.

Step 2: This step makes use of IDs $f_{ID}(\xi_e|\xi_{e'<e},\theta,y)$, which approximate the univariate distributions $\pi(\xi_e|\xi_{e'<e},\theta,y)$ for e=1,…,E (with functional form chosen to provide good approximation to the model under study). Each functional form for the IDs is associated with a different (posterior based) proposal distributions $g(\xi_e^p)$ satisfying Eq.(3.4) (see Table 1). IDs are characterised by one or two parameters (*e.g.* an expected event number in the Poisson case and a mean and variance in the normal case) and for e>1 these functionally depend on latent variables with lower index (as determined by the DAG structure of the underlying the model) and the model parameters and data.

Step 3: Two limiting cases in Eq.(3.7) are of particular importance. Firstly, as $f_{ID}(\xi_e|\xi_{e'<e},\theta,y)$ becomes more and more representative of $\pi(\xi_e|\xi_{e'<e},\theta,y)$, so the MH probability reduces to

$$P_{MH} = \min\left\{1, \frac{\pi(\theta^p|y)}{\pi(\theta^i|y)}\right\}. \tag{E1}$$

[4] In other words, starting at θ^p and ξ^p and proposing the state θ^i and ξ^i.

[5] The probability of jumping from θ^i to θ^p is the same as from θ^p to θ^i.

The originally high dimensional problem (containing latent variables ξ and parameters θ) is reduced to a much lower dimensional problem (containing just parameters θ), helping explain why mixing is potentially so much faster.

Secondly, as the jumping size in parameter space gets smaller (as determined by *j* in Eq.(3.6)) so $P_{MH}\rightarrow 1$. This is of particular importance because it means that even if the IDs provide a relatively poor approximation to $\pi(\xi_e|\xi_{e'<e},\theta,y)$, provided *j* is made sufficiently small a substantial fraction of proposals will always be accepted. In practice the jumping size *j* is optimally tuned to ensure acceptance around 33% of the time (see appendix C for details).

Appendix F: Other possibilities for proposals in parameter space

Three issues are discussed in relation to proposals in parameter space:

1) Optimisation

The proposal distribution in parameter space introduced in Eq.(3.6) has the advantage of being simple and robust against highly correlated model parameters. Generally speaking, however, it may not represent the optimum choice. For example, if two variables A and B are largely uncorrelated in the posterior it may actually be computationally faster to consider proposals to A and B separately. This is especially true in cases when proposing changes to A is much faster (*e.g.* fixed effects in mixed models) than B (*e.g.* random effects).

In the most general case, a combination of the following two types of proposal can be used in Eq.(3.6):

Single parameters changes – A single parameter *k* is selected from θ. θ^p_k is then sampled from a simple normal distribution centred at θ^i_k:

$$\theta^p_k \sim N(\theta^i_k, \sigma^2_k), \quad \theta^p_{l\neq k} = \theta^i_l. \tag{F1}$$

Multiple parameter changes – χ represent a subset of the parameters in θ, and θ^p_χ is sampled from a multivariate normal distribution centred at θ^i_χ:

$$\theta^p_\chi \sim N(\theta^i_\chi, j^2_\chi \Sigma^\chi), \quad \theta^p_{l\notin\chi} = \theta^i_l. \tag{F2}$$

Providing an automated way to determine the optimum choice for proposals in parameter space for a given model will be the subject of future research.

2) Parameters not continuous

In some cases model parameters are discrete, *e.g.* for an epidemiological model the initial population numbers in various compartments might be included in θ. If these discrete variables are approximately normally distributed (as they would be if the population sizes are reasonably large), then we could again draw a vector *v* from a MVN distribution as before

$$v \sim N(\theta^i, j^2\Sigma^\theta), \tag{F3}$$

but this time round those discrete model variables to the nearest integer

$$\theta_k^p = \begin{cases} \text{round}(v_j), & \text{if } \theta_j \text{ discrete} \\ v_j, & \text{if } \theta_j \text{ continuous} \end{cases}. \tag{F4}$$

In cases in which model parameters are not expected to be approximately normally distributed they can simply be updated individually.

3) Restrictions

For some of the functional forms in Table 1, only one of the characteristic parameters can be updated at a time. For example, for the beta distribution only α can be changed whilst fixing β, or *vice versa*. Consequently, proposals to both α and β cannot be performed simultaneously.

Appendix G: Normal distributions

The probability density function for drawing a value x from a normal distribution with mean μ and variance σ^2 is given by

$$f_{\text{norm}}(x \mid \mu, \sigma^2) = \frac{1}{\sqrt{2\pi\sigma^2}} e^{-\frac{(x-\mu)^2}{2\sigma^2}}. \tag{G1}$$

The equivalent multivariate normal distribution is

$$f_{MVN}(\boldsymbol{x} \mid \boldsymbol{\mu}, \boldsymbol{\Sigma}) = \frac{1}{\sqrt{(2\pi)^d |\Sigma|}} e^{-\frac{1}{2}(\boldsymbol{x}-\boldsymbol{\mu})^T \boldsymbol{\Sigma}^{-1} (\boldsymbol{x}-\boldsymbol{\mu})}, \tag{G2}$$

where d is the number of dimensions and $\boldsymbol{\Sigma}$ is the covariance matrix that captures the variance of individual elements (*e.g.* parameters) as well as covariance between them. Cholesky decomposition provides a standard way to draw samples from a multivariate normal distribution [5]. Provided $\boldsymbol{\Sigma}$ is positive-definite it can be written as $\boldsymbol{\Sigma}=\boldsymbol{B}\boldsymbol{B}^T$, where $\boldsymbol{B}$ is a lower triangular matrix. Samples from the multivariate normal are then generated using

$$\boldsymbol{x} = \boldsymbol{\mu} + \boldsymbol{B}\boldsymbol{z}, \tag{G3}$$

where $\boldsymbol{z}$ is a vector of normally distributed independent samples with mean zero and unit variance.

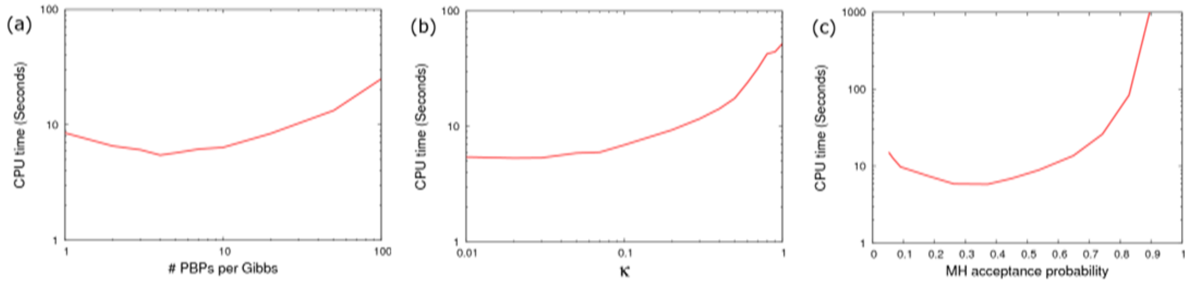

Figure H: Optimisation of key parameters.

Appendix H: Optimisation of the PBP MCMC algorithm

Figure H illustrates how the PBP algorithm is typically optimised. These results are based on the mixed model applied to quantitative genetics introduced in §5.3, but the general findings are found to be largely independent of model type. Optimisation can be considered from three points of view:

Firstly, Fig. H(a) shows the CPU time to generate 100 effective samples of r^2 from the posterior as a function of the number of PBPs between each Gibbs update of the latent variables. We find that this particular curve has a minimum at U=4 updates, although computation speed is found to not be particularly sensitive to its exact value.

Secondly, Fig. H(b) shows variation in CPU time as a function of the tuneable constant κ (this is used in Table 1 in cases in which the ID is normally distributed). Again, performance is largely the same provided κ is smaller than around 0.1. For this study κ=0.03 was selected.

Finally, Fig. H(c) shows that the algorithm is optimised when the MH acceptance probability is around 33%. This is implemented using the methods outlined in appendix C.

Appendix I: Non-centred parameterisations

It has long been recognized that the parametrization of hierarchical models can be crucial for MCMC performance [6]. A so-called "centred" parameterisation (CP) is the default option given by the specification of the model in terms of parameters θ which determine the distribution of latent variables ξ. On the other hand a "non-centred" parameterisation (NCP) refers to the case in which a new set of latent variables ξ' are defined so as to be distributed conditionally independently of θ, and the original latent variables are functionally dependant on these, *i.e.* ξ=*h*(ξ',θ,y).

To give a simple example, suppose each latent variable is distributed normally $\xi_e \sim N(\mu, \sigma^2)$ with mean μ and variance σ^2 being model parameters θ. This can be reparametrized by setting $\xi'_e \sim N(0,1)$, with the functional dependency *h* being given by $\xi_e = \mu + \sigma\xi'_e$.

Appendix J: Hamiltonian MCMC

The reason standard approaches (involving small local changes) are slow is because they behave diffusively. One proposal might move a parameter in one direction, but the next might move it back again to near where it started. Such random walk behaviour often leads to slow progress from one side of the posterior to the other, which is especially true for high dimensional problems. The idea behind HMCMC is to make large jumps in parameter space to overcome this diffusive behaviour.

HMCMC [7, 8] makes no distinction between model parameters and latent variables, and so subsequently we refer to the combination $\boldsymbol{x}$=(θ,ξ) to represent a vector giving the overall parameters in the model. The intuition behind HMCMC comes from physics. We first define $U(\boldsymbol{x})$=-log($\pi(y|\boldsymbol{x})\pi(\boldsymbol{x})$) as the negative log of the posterior probability, where $U(\boldsymbol{x})$ maps out a potential energy landscape, and consider a particle moving in this space. The particle has both a position vector $\boldsymbol{x}$ and a momentum vector $\boldsymbol{p}$. Just as a ball on a hill runs down and accelerates, so a particle with high potential U gets pushed towards lower U, at the same time increasing its kinetic energy. An important principle in physics is the conservation of energy. Here we defined the total energy of the system by the Hamiltonian

$$H(\boldsymbol{x},\boldsymbol{p}) = \tfrac{1}{2}\boldsymbol{p}^T\boldsymbol{M}^{-1}\boldsymbol{p} + U(\boldsymbol{x}), \tag{J1}$$

where the first term represents the kinetic energy (***M*** is the mass matrix, as identified below) and the second term represents the potential energy.

The following algorithm outlines the procedure for a single HMCMC update.

HMCMC algorithm

Step 1: Sample momentum – The initial momentum vector at time t=0 is sampled according to

$$\boldsymbol{p}(0) \sim N(0,\boldsymbol{M}), \tag{J2}$$

and $\boldsymbol{x}(0)$ is set to the current parameter set $\boldsymbol{x}^i$ on the MCMC chain.

Step 2: Integration of trajectory – The following leapfrog algorithm is iterated L times:

$$\begin{aligned}\boldsymbol{p}(t+\tfrac{\varepsilon}{2}) &= \boldsymbol{p}(t) - \frac{\varepsilon}{2}\nabla_x U\big|_t,\\ \boldsymbol{x}(t+\varepsilon) &= \boldsymbol{x}(t) + \varepsilon\boldsymbol{M}^{-1}\boldsymbol{p}(t+\tfrac{\varepsilon}{2}),\\ \boldsymbol{p}(t+\varepsilon) &= \boldsymbol{p}(t+\tfrac{\varepsilon}{2}) - \frac{\varepsilon}{2}\nabla_x U\big|_{t+\varepsilon},\end{aligned} \tag{J3}$$

where ε is the integration step size and $\boldsymbol{\nabla}_x U|_t$ is the gradient in the potential energy evaluated at time t (note, this vector points uphill in the potential energy landscape)**.** This procedure represents a numerical approximation to Hamilton's equations.

Step 3: Accept or reject – The final proposed state $\boldsymbol{x}^p$=$\boldsymbol{x}(L\varepsilon)$ is accepted or rejected with MH probability functionally dependent on the difference in the Hamiltonian between the initial and final states:

$$P_{MH} = \min\left\{1, e^{H(\boldsymbol{x}(0),\boldsymbol{p}(0)) - H(\boldsymbol{x}(L\varepsilon),\boldsymbol{p}(L\varepsilon))}\right\}. \tag{J4}$$

Note because the Hamiltonian is conserved, Eq.(J4) is expected to be near to one. The reason it isn't exactly one is because the continuous integral is numerically approximated by the discrete leapfrog method (consequently $P_{MH}\rightarrow 1$ as $\varepsilon\rightarrow 0$, but if ε is large P_{MH} can become very small if a long trajectory is integrated over).

Optimisation

HMCMC is most efficient when the inverse of the mass matrix $\boldsymbol{M}^{-1}$ is given by a numerical approximation to the covariance matrix for the posterior distribution $\pi(\boldsymbol{x}|y)$. However in high dimensional situations this is usually too computationally demanding to calculate (*e.g.* matrix inversion takes of order D^3 operations, where D is the number of dimensions). Instead two possibilities are commonly implemented: either set ***M*** to the identity matrix or set it to be diagonal with elements given by the inverse of the posterior parameter variances. Here we chose the latter option, as this was found to improve mixing times compared to the former.

The algorithm above contains two tuneable parameters: step size ε and number of steps *L*. Optimising the step size ε is relatively easy, as it can be selected to achieve a certain average acceptance rate. If ε is very small then the acceptance probability will be almost one but computation will be slowed down because more and more intermediary steps will be needed for a certain integration length *Lε*. On the other hand if ε is too large, most proposals get rejected. The optimal acceptance rate (under some strong assumptions) has been shown to be approximately 0.65 [9], which is used here (although efficiency was not found to be very sensitive to this precise value).

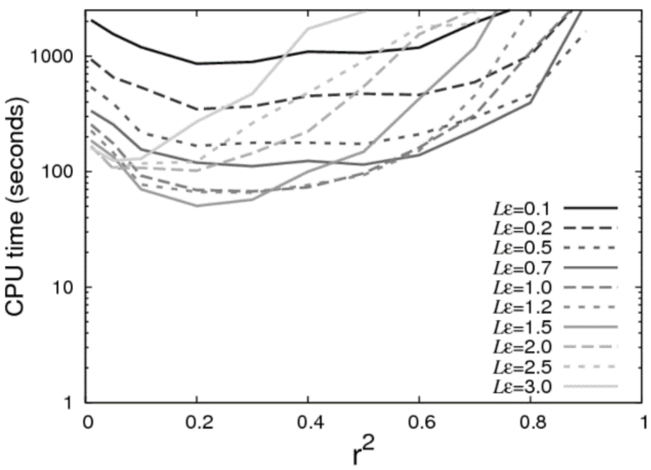

Figure J: Optimisation of HMCMC. These results are applicable to the mixed model in §5.3 and shows how the CPU time needed to generate 100 effective samples of r^2 (which characterises the genetic heritability) varies as the r^2 used to simulate the data changes. Each of the curves corresponds to running NCP HMCMC using different fixed integration lengths *L*ε (ε is adaptively tuned to give an acceptance probability of 0.65). The curve defined by the lowest points in this diagram represents the optimised NCP HMCMC results, as shown by the green dashed line in Fig.7(b).

Optimising the number of steps for each update *L*, however, is difficult and efficiency is found to critically depend on this value. Automated methods such as the No U-Turn Sampler (NUTS) [9] have been developed, but these are challenging to implement. This paper takes a brute force approach to find the optimal HCMC implementation. For each set of simulated data, inference is carried out using a large number of different values of integral length *Lε*. The most efficient of these is used to construct the HMCMC curves in Figs. 6(d) and 7(b). An example of this process is shown in Fig. J, which demonstrates how the NCP HMCMC results in Fig. 7(b) were generated. Note, under realistic models the optimal results from NUTS were found to have a very similar computational efficiency to HMCMC tuned in this fashion [9].

Appendix K: Particle MCMC

The idea behind PMCMC is that random walk MCMC can be run on the basis of an unbiased approximation to $\pi(y|\theta)$. Because the dimensionality of θ is typically much less than ξ, this algorithm is expected to mix at a much faster rate than standard MCMC. The drawback of this approach, however, is that obtaining a sufficiently accurate estimate $\hat{\pi}(y|\theta)$ for $\pi(y|\theta)$ can be computationally demanding.

The algorithm below describes the implementation used in this paper [10][6] :

[6] Note, this method is known as the "particle marginal Metropolis–Hastings" (PMMH) sampler in this reference. The proposals in parameter space in Eq.(K1) are chosen to be consistent with Eq.(3.6) to allow for fair comparison between methods.

PMCMC algorithm

Step 1: Generate θ^p – This is the same as for PBPs. A proposed set of parameter values is drawn from a multivariate normal (MVN) distribution centred on the current set of parameters in the chain θ^i

$$\theta^p \sim N(\theta^i, j^2\Sigma^\theta), \tag{K1}$$

where Σ^θ is a numerical approximation to the covariance matrix for $\pi(\theta|y)$ and *j* is a tuneable jumping parameter (estimation of Σ^θ and optimisation of *j* are achieved during an initial "adaptation" period, as explained in appendix C).

Step 2: Generate unbiased estimate $\hat{\pi}(y|\theta^p)$ – We take each latent variable ξ_e in turn (starting from *e=1* up to *e=E*) and consider *Z* particles. The weights for these particles are initially set to w_z=1. For each particle *z* we sample from an importance distribution

$$\xi_e^z \sim f_{ID}(\xi_e^z \,|\, \xi_{e'<e}^z, \theta^p, y). \tag{K2}$$

In the simplest case this will be ID_0, which is equivalent to simulating from the model, but as with PBPs, greater efficiency can be achieved by using higher order importance distributions. Here we imagine the case in which an observation y_e is made on each latent variable with observation probability $\pi(y_e|\xi_e,\theta)$. The weight for each particle w_z is then multiplied by

$$\pi(y_e \,|\, \xi_e^z, \theta^p)\frac{\pi(\xi_e^z \,|\, \xi_{e'<e}^z, \theta^p)}{f_{ID}(\xi_e^z \,|\, \xi_{e'<e}^z, \theta^p, y)}. \tag{K3}$$

After scanning through all latent variables, an unbiased estimator can be generated by

$$\pi(y \,|\, \theta^p) = \tfrac{1}{Z}\sum_{z=1}^{Z} w_z, \tag{K4}$$

which is essentially a standard implementation of importance sampling (see appendix A). This estimator, however, turns out to usually be very computationally wasteful because most of the particles have almost zero weight and contribute very little to the sum. A key innovation in PMCMC is the introduction of "bootstrap" steps. At various points when scanning from *e*=1 to *E*, a new set of particles is sampled from the existing set with probability proportional to the particle weights. This new set then has its weights returned back to w_z=1 and the process is continued. Now

$$\pi(y \,|\, \theta^p) = \prod_b \left(\tfrac{1}{Z}\sum_{z=1}^{Z} w_z^b\right) \tag{K5}$$

is an unbiased estimator, where the product goes over bootstrap steps and w_z^b are the weights of particles immediately prior to the bootstrap being performed.

Step 3: Accept or reject – The final proposed state θ^p is accepted or rejected with MH probability

$$P_{MH} = \min\left\{1, \frac{\pi(y \,|\, \theta^p)\pi(\theta^p)}{\pi(y \,|\, \theta^i)\pi(\theta^i)}\right\}. \tag{K6}$$

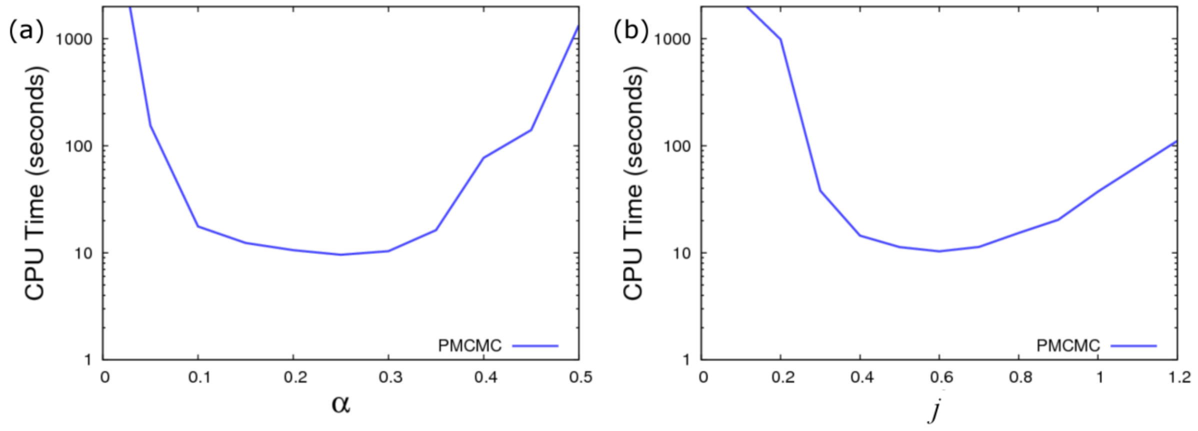

Figure K: Optimisation of the PMCMC algorithm. Results are shown for the logistic population model in §5.4 (CPU time when 3 population measurements are made). Shows how CPU time for 100 effective samples of r_b varies as a function of (a) the average acceptance rate α (fixing j=0.6) and (b) the parameter jumping size j (fixing α=0.25).

Optimisation

The PMCMC algorithm above has two free parameters which need to be optimised: the jumping size j used in Eq.(K1) and the number of particles Z. The former can be fixed to an optimised value and the latter can be tuned to give a certain specified acceptance rate α. This is achieved in the algorithm by introducing a floating point version of the particle number Z_f (such that Z is the integer rounded value of Z_f) which is updated in the following manner:

$$\begin{aligned} Z_f^{new} &= Z_f \times 1.02 && \text{if PMCMC proposal accepted,} \\ Z_f^{new} &= Z_f \times 1.02^{-\frac{\alpha}{1-\alpha}} && \text{if PMCMC proposal rejected.} \end{aligned} \quad \text{(K7)}$$

(note, this is analogous to the approach used in Eqs.(C2) and (C3)).

Figure K shows the algorithm can be optimised by scanning j and α. In this particular example CPU is minimised when α≈0.25 and j≈0.6, with performance not very sensitive to these precise values.

Appendix L: Effective sample number

Given X correlated MCMC samples of some quantity x^i, the autocorrelation function can be approximated by

$$F_\tau = \frac{1}{(X-\tau)\overline{\sigma_x^2}} \sum_{i=1}^{X-\tau} \left(x^i - \bar{x}\right)\left(x^{i+\tau} - \bar{x}\right), \quad \text{(L1)}$$

where estimates for the average and variance of x are given by

$$\bar{x} = \tfrac{1}{X} \sum_{i=1}^{X} x^i, \quad \overline{\sigma_x^2} = \frac{1}{X-1} \sum_{i=1}^{X} \left(x^i - \bar{x}\right)^2. \quad \text{(L2)}$$

The effective sample size is given by the actual sample number X, correcting for correlations between successive samples:

$$X_{eff} = \frac{X}{1+2\sum_{\tau=1}^{\infty} F_\tau}. \quad \text{(L3)}$$

When actually calculating X_{eff}, clearly the sum in Eq.(L3) cannot go to infinity. In fact, F_τ often exhibits considerable fluctuations for large τ, and these can generate unwanted bias. The simplest way to deal with these is to truncate the sum in Eq.(L3) up to a maximum size $\tau_{\max}$, which is defined to be the largest value of τ for which the following condition holds true (see [11]):

$$F_\tau > 0.05. \quad \text{(L4)}$$

Appendix M: Details for disease prevalence model

In this appendix we provide additional details relevant to the disease prevalence and diagnostic test model in §5.1.

Simulation and prior details

Simulated data was created using Se_1=Se_2=0.6 and Sp_1=Sp_2=0.9 for P=1000 individuals. Different values of individual number P were used to generate Fig. 5(d). The prior distributions for parameters Se_1, Se_2 and p_D were assumed to be uniform between 0 and 1, and for Sp_1 and Sp_2 to be uniform between 0.5 and 1 (the reason this isn't from 0 is because otherwise the posterior becomes bimodal).

Observation model and latent process likelihood

The model is illustrated in Fig. 5(a). The true disease status of individuals is represented by Bernoulli variables D_e, where D_e=1 (or 0) denotes that individual e is infected (or uninfected) with probability p_D (or 1-p_D). The test data y_e^t for test type t are 1 (or 0), indicating a positive (or negative) result.

We identify the following model parameters θ={p_D,Se_1,Sp_1,Se_2,Sp_2} and latent variables ξ={D}. The observation model and latent process likelihood are given by

$$\begin{aligned} \pi(y \mid \xi,\theta) &= \prod\nolimits_{t=1,2} Se_t^{N_t^{1|1}} \left(1-Se_t\right)^{N_t^{0|1}} Sp_t^{N_t^{0|0}} \left(1-Sp_t\right)^{N_t^{1|0}}, \\ \pi(\xi \mid \theta) &= p_D^{N^1} \left(1-p_D\right)^{N^0}, \end{aligned} \quad \text{(M1)}$$

where N^d is the number of individuals with disease status d and $N_t^{r|d}$ is the number of cases in which test t gives result r for individuals with disease status d.

Importance distributions

Step 2 of the PBP algorithm (introduced in §3.4) makes use of IDs. Successive approximations for these IDs are discussed in §4. Here we explicitly present expressions for this particular model.

ID_0 is given by the model itself

$$f_{ID_0}(D_e \mid D_{e'<e},\theta) = f_{Bern}(D_e \mid p_D), \quad \text{(M2)}$$

where f_{Bern} is the Bernoulli probability distribution.

ID$_1$ takes into account both the model and the observations. From Eq.(4.7) this is given by

$$f_{ID_1}(D_e \mid D_{e'<e}, \theta, y) = c f_{Bern}(D_e \mid p_D) \times \prod_{t=1,2} \pi(y_e^t \mid D_e), \tag{M3}$$

where *c* is a normalisation constant. Explicitly incorporating the observation model from Eq.(M1), this becomes

$$f_{ID_1}(D_e \mid D_{e'<e}, \theta, y) = f_{Bern}(D_e \mid \tfrac{p_1}{p_1+p_0}), \tag{M4}$$

where

$$\begin{aligned} p_1 &= p_D \times \begin{cases} Se_1 \text{ if obs. 1 +ve} \\ 1-Se_1 \text{ if obs. 1 -ve} \end{cases} \times \begin{cases} Se_2 \text{ if obs. 2 +ve} \\ 1-Se_2 \text{ if obs. 2 -ve} \end{cases} \\ p_0 &= (1-p_D) \times \begin{cases} 1-Sp_1 \text{ if obs. 1 +ve} \\ Sp_1 \text{ if obs. 1 -ve} \end{cases} \times \begin{cases} 1-Sp_2 \text{ if obs. 2 +ve} \\ Sp_2 \text{ if obs. 2 -ve} \end{cases}. \end{aligned} \tag{M5}$$

In this particular example ID$_1$ (unusually) represents the exact importance distribution (*i.e*. it directly samples from the posterior), so no higher order terms need to be considered.

Proposals

As an illustration of how PBPs are implemented in practice, we explicitly go through step 2 of the PBP algorithm from §3.4 (which stochastically modifies D_e^i to generate D_e^p).

The ID is given by a Bernoulli distribution with disease probability *z*:

$$f_{ID}(D_e \mid D_{e'<e}, \theta, y) = f_{Bern}\left(D_e \mid z\right). \tag{M6}$$

For ID$_0$, *z* is equal to p_D and for ID$_1$, $z=p_1/(p_1+p_0)$, where the definitions for p_0 and p_1 are given in Eq.(M5).

Sequentially going through each individual *e*, the values for the initial z_e^i and proposed z_e^p states are calculated. Table 1 shows that for the Bernoulli distribution:

1) For $z_e^p > z_e^i$: if $D_e^i = 1$ we simply set $D_e^p = 1$, otherwise we draw a random number from *0* to *1* and if it is less than $\frac{z_e^p - z_e^i}{1-z_e^i}$ we set $D_e^p = 1$ else $D_e^p = 0$.

2) For $z_e^p \le z_e^i$: if $D_e^i = 0$ we simply set $D_e^p = 0$, otherwise we draw a random number from *0* to *1* and if it is less than $1-\frac{z_e^p}{z_e^i}$ we set $D_e^p = 0$ else $D_e^p = 1$.

Gibbs samplers

For the disease diagnostic test model it is possible to explicitly sample directly from the posterior when model parameters and latent variables are each considered separately.

Model parameters: The following samples are sequentially drawn from beta distributions

$$
\begin{aligned}
p_D &\sim Beta(N^1+1, N^0+1), \\
Se_1 &\sim Beta(N_1^{1|1}+1, N_1^{0|1}+1), \\
Sp_1 &\sim Beta(N_1^{0|0}+1, N_1^{1|0}+1), \\
Se_2 &\sim Beta(N_2^{1|1}+1, N_2^{0|1}+1), \\
Sp_2 &\sim Beta(N_2^{0|0}+1, N_2^{1|0}+1).
\end{aligned} \tag{M7}
$$

In the case of Sp_1 and Sp_2 samples are rejected if less than 0.5 (to respect the prior), but this occurs very infrequently.

Latent variables: Each individual *e* is considered in turn and, using the definitions in Eq.(M5), we set $D_e=1$ with probability $p_1/(p_1+p_0)$ else $D_e=0$.

Appendix N: Details of the stochastic volatility model

In this appendix we provide additional details relevant to stochastic volatility model in §5.2.

Simulation and prior details

Simulated data was created using μ=-10, ϕ=0.99, ν=12, σ^2=0.0121, and for simplicity the initial condition was set to $h_1=\mu$. Different value of correlation parameter ϕ were used to generate Fig. 6(d). The prior distributions for all variables were taken to be flat and in the ranges -∞–∞ for μ and h_1, 0.0001–0.9999 for ϕ, 2–50 for ν and 0–∞ for σ^2.

Observation model and latent process likelihood

We identify the following model parameters $\theta=\{\mu,\phi,\nu,\sigma^2,h_1\}$ and latent variables $\xi=\{h_{e>1}\}$. The DAG structure is illustrated in Fig. 6(a). The observation model and latent process likelihood are given by

$$
\begin{aligned}
\pi(y|\xi,\theta) &= \prod_{e=1}^{E} \frac{\Gamma(\frac{\nu+1}{2})}{\sqrt{\nu\pi}\Gamma(\frac{\nu}{2})}\left(1+\frac{y_e^2}{\nu e^{h_e}}\right)^{-\frac{\nu+1}{2}} e^{-h_e/2}, \\
\pi(\xi|\theta) &= \prod_{e=2}^{E} \frac{1}{\sqrt{2\pi\sigma^2}} e^{-\frac{(h_e-\mu-\phi(h_{e-1}-\mu))^2}{2\sigma^2}},
\end{aligned} \tag{N1}
$$

where Γ is the gamma function and *E* the observation period.

Importance distributions

ID_0 is given by the model

$$
f_{ID_0}(h_e|h_{e'<e},\theta) = f_{\text{norm}}\left(h_e|\mu_e^{\text{mod}},\sigma^2\right), \tag{N2}
$$

where $\mu_e^{\text{mod}} = \mu+\phi\left(h_{e-1}-\mu\right)$.

Equation (4.7) shows the expression for ID_1. The first thing to note is that the product of the model $\pi(\xi_e|\xi_{e'<e},\theta)$ and observation probability $\pi(y_e|\xi_e,\theta)$ distributions from Eq.(N1) is not a standard distribution with which PBPs can be used (*i.e.* it is not listed in Table 1). One way around this problem is to first approximate the observation model as a normal distribution. This is achieved by Taylor series expanding $\log(\pi(y_e|\xi_e,\theta))$ about μ_e^{mod} up to second order, leading to

$$\pi(y_e \mid \xi_e, \theta) \cong f_{\text{norm}}\left(h_e \mid \mu_e^{\text{obs}}, \sigma_e^{\text{obs}2}\right), \tag{N3}$$

where

$$\mu_e^{\text{obs}} = \mu_e^{\text{mod}} + \tfrac{1}{2}\sigma_e^{\text{obs}2}\frac{\nu - q_e}{q_e+1}, \quad \sigma_e^{\text{obs}2} = \frac{2(q_e+1)^2}{q_e(\nu_e+1)}, \quad q_e = \frac{\nu e^{\mu_e^{\text{mod}}}}{y_e^2}. \tag{N4}$$

The product of the two normal distributions in Eqs.(N2) and (N3) give

$$f_{ID_1}(h_e \mid h_{e'<e}, \theta, y) = f_{\text{norm}}\left(h_e \mid \tfrac{\mu_e^{\text{obs}}\sigma^2 + \mu_e^{\text{mod}}\sigma_e^{\text{obs}2}}{\sigma_e^{\text{obs}2}+\sigma^2}, \tfrac{\sigma_e^{\text{obs}2}\sigma^2}{\sigma_e^{\text{obs}2}+\sigma^2}\right). \tag{N5}$$

The definition of ID_2 from Eq.(4.8) is given by

$$f_{ID_2}(h_e \mid h_{e'<e}, \theta, y) \propto \pi(h_e \mid h_{e-1}, \theta)\pi(y_e \mid h_e, \theta)\int \pi(h_{e+1} \mid h_e, \theta)\pi(y_{e+1} \mid h_{e+1}, \theta)\mathrm{d}h_{e+1}. \tag{N6}$$

In other words, the posterior distribution for h_e at time e depends not only on the value of h_{e-1} (*i.e.* the previous time point), but also on the observations at times e and $e+1$. In the general case this integral is intractable, but again following the Taylor series expansion approximation to the observation likelihood around $\bar{\mu} = \mu_e^{\text{mod}} = \mu + \phi\left(h_{e-1} - \mu\right)$, the following set of approximations can be made:

$$\begin{aligned}
&\pi(h_e \mid h_{e-1}, \theta) = f_{\text{norm}}\left(h_e \mid \bar{\mu}, \sigma^2\right), \\
&\pi(y_e \mid h_e, \theta) \cong f_{\text{norm}}\left(h_e \mid \mu_e^{\text{obs}}, \sigma_e^{\text{obs}2}\right), \\
&\pi(h_{e+1} \mid h_e, \theta) = f_{\text{norm}}\left(h_{e+1} \mid \mu + \phi\left(h_e - \mu\right), \sigma^2\right), \\
&\pi(y_{e+1} \mid h_{e+1}, \theta) \cong f_{\text{norm}}\left(h_{e+1} \mid \mu_{e+1}^{\text{obs}}, \sigma_{e+1}^{\text{obs}2}\right).
\end{aligned} \tag{N7}$$

where

$$\begin{aligned}
&\mu_e^{\text{obs}} = \bar{\mu} + \tfrac{1}{2}\sigma_e^{\text{obs}2}\frac{\nu - q_e}{q_e+1}, \quad \sigma_e^{\text{obs}2} = \frac{2(q_e+1)^2}{q_e(\nu_e+1)}, \quad q_e = \frac{\nu e^{\bar{\mu}}}{y_e^2}, \\
&\mu_{e+1}^{\text{obs}} = \bar{\mu} + \tfrac{1}{2}\sigma_{e+1}^{\text{obs}2}\frac{\nu - q_{e+1}}{q_{e+1}+1}, \quad \sigma_{e+1}^{\text{obs}2} = \frac{2(q_{e+1}+1)^2}{q_e(\nu_{e+1}+1)}, \quad q_{e+1} = \frac{\nu e^{\bar{\mu}}}{y_{e+1}^2}.
\end{aligned} \tag{N8}$$

Substituting the results from Eq.(N7) into (N6) gives

$$\begin{aligned}
f_{ID_2}(h_e \mid h_{e'<e}, \theta, y) \propto\ & f_{\text{norm}}\left(h_e \mid \bar{\mu}, \sigma^2\right) f_{\text{norm}}\left(h_e \mid \mu_e^{\text{obs}}, \sigma_e^{\text{obs}2}\right) \times \\
& \int f_{\text{norm}}\left(h_{e+1} \mid \mu + \phi\left(h_e - \mu\right), \sigma^2\right) f_{\text{norm}}\left(h_{e+1} \mid \mu_{e+1}^{\text{obs}}, \sigma_{e+1}^{\text{obs}2}\right)\mathrm{d}h_{e+1}.
\end{aligned} \tag{N9}$$

Integrating over two normally distributed quantities is Gaussian distributed with respect to the difference in means with variance given by the sum of the variances of the two original distributions. Consequently, Eq.(N9) becomes

$$f_{ID_2}(h_e \mid h_{e'<e},\theta,y) \propto f_{\text{norm}}\left(h_e \mid \overline{\mu},\sigma^2\right) f_{\text{norm}}\left(h_e \mid \mu_e^{\text{obs}},\sigma_e^{\text{obs}2}\right) \times e^{-\frac{\left(\mu+\phi\left(h_e-\mu\right)-\mu_{e+1}^{\text{obs}}\right)^2}{2\left(\sigma^2+\sigma_{e+1}^{\text{obs}2}\right)}}. \quad \text{(N10)}$$

When written in terms of h_e the last term becomes another normal distribution

$$f_{ID_2}(h_e \mid h_{e'<e},\theta,y) \propto f_{\text{norm}}\left(h_e \mid \overline{\mu},\sigma^2\right) f_{\text{norm}}\left(h_e \mid \mu_e^{\text{obs}},\sigma_e^{\text{obs}2}\right) f_{\text{norm}}\left(h_e \mid \mu_e^{next},\sigma_e^{next2}\right), \quad \text{(N11)}$$

with mean and variance that capture information from the next observation (*i.e.* at time *e*+1)

$$\mu_e^{next} = \mu + \frac{\mu_{e+1}^{\text{obs}}-\mu}{\phi}, \qquad \sigma_e^{next2} = \frac{\sigma^2+\sigma_{e+1}^{\text{obs}2}}{\phi^2}. \quad \text{(N12)}$$

The product of the three normal distributions in Eq.(N11) gives the final result

$$f_{ID_2}(h_e \mid h_{e'<e},\theta,y) = f_{\text{norm}}(h_e \mid \mu_e^{res},\sigma_e^{res2}), \quad \text{(N13)}$$

where

$$\frac{1}{\sigma_e^{res2}} = \frac{1}{\sigma^2} + \frac{1}{\sigma_e^{\text{obs}2}} + \frac{1}{\sigma_e^{next2}}, \qquad \frac{\mu_e^{res}}{\sigma_e^{res2}} = \frac{\overline{\mu}}{\sigma^2} + \frac{\mu_e^{\text{obs}}}{\sigma_e^{\text{obs}2}} + \frac{\mu_e^{next}}{\sigma_e^{next2}}. \quad \text{(N14)}$$

The standard approach

By multiplying the two expressions in Eq.(N1), the posterior distribution is given by

$$\pi(\mu,\phi,\nu,\sigma^2,\boldsymbol{h} \mid y) \propto \prod_{e=2}^{E} \frac{1}{\sqrt{2\pi\sigma^2}} e^{-\frac{\left(h_e-\mu-\phi\left(h_{e-1}-\mu\right)\right)^2}{2\sigma^2}} \times \prod_{e=1}^{E} \frac{\Gamma(\frac{\nu+1}{2})}{\sqrt{\nu\pi}\,\Gamma(\frac{\nu}{2})} \left(1+\frac{y_e^2}{\nu e^{h_e}}\right)^{-\frac{\nu+1}{2}} e^{-h_e/2}. \quad \text{(N15)}$$

Rearranging this gives

$$\pi(\mu \mid y,\phi,\nu,\sigma^2,\boldsymbol{h}) \propto e^{-\frac{(1-\phi)^2(\text{E}-1)\left(\mu-\frac{1}{(1-\phi)(E-1)}\sum_{e=2}^{E} h_e-\phi h_{e-1}\right)^2}{2\sigma^2}}, \quad \text{(N16)}$$

which takes the form of a standard normal distribution

$$\pi(\mu \mid y,\phi,\nu,\sigma^2,\boldsymbol{h}) = f_{\text{norm}}\left(\mu \mid \frac{1}{(1-\phi)(E-1)}\sum_{e=2}^{E} h_e-\phi h_{e-1}, \frac{\sigma^2}{(1-\phi)^2(\text{E}-1)}\right). \quad \text{(N17)}$$

Consequently, μ is sampled in the following way:

$$\mu \sim N\left(\frac{1}{(1-\phi)(E-1)}\sum_{e=2}^{E} h_e-\phi h_{e-1}, \frac{\sigma^2}{(1-\phi)^2(\text{E}-1)}\right). \quad \text{(N18)}$$

Similarly, the correlation parameter ϕ is also sampled from a normal distribution

$$\phi \sim N\left(\frac{\sum_{e=2}^{E}\left(h_e-\mu\right)\left(h_{e-1}-\mu\right)}{\sum_{e=2}^{E}\left(h_{e-1}-\mu\right)^2}, \frac{\sigma^2}{\sum_{e=2}^{E}\left(h_{e-1}-\mu\right)^2}\right). \quad \text{(N19)}$$

The expression in Eq.(N15) can be rearranged to give

$$\pi(\sigma^2 \mid y,\mu,\phi,\nu,\boldsymbol{h}) \propto \frac{1}{\sigma^{E-1}} e^{-\frac{\sum_{e=2}^{E}(h_e-\mu-\phi(h_{e-1}-\mu))^2}{2\sigma^2}}, \tag{N20}$$

which is an inverted chi-squared distribution with respect to σ^2. All samples generated that have zero prior probability are rejected.

We adopt a simple random walk Metropolis-Hastings scheme (*i.e.* propose a new parameter / variable by adding a normally distributed contribution to its existing value and accepting or rejecting that change) for ν and h_e. In the case of h_e care is taken to only calculate those parts of the latent process likelihood and observation probability that actually change (to optimise the code as far as possible). The jumping sizes of the separate proposals are individually tuned to give acceptance approximately 33% of the time (using the same procedure as for *j* in appendix C).

Appendix O: Details for mixed models

In this appendix we provide additional details relevant to the mixed model in §5.3.

Simulation and prior details

In all cases flat uninformative priors were assumed for parameters.

Here we take $\boldsymbol{y}$ to represent measurements of heights within a population. Two fixed effects are assumed: β_1 represents the average height of females and β_2 gives the average height difference between males and females. As illustrated in Fig. O1, the model assumes a population of size *P* randomly mated over four generations (which leads to a sparse inverse matrix $\boldsymbol{A}^{-1}$ [7]). Here individuals in the 1st generation are assumed to be unrelated (*i.e.* conditionally independent) and those in the 2nd, 3rd and 4th generations are conditionally dependent on exactly two individuals in the previous generation (*i.e.* their parents). Simulated data was generated using a population size of *P*=1000, two fixed effects $\boldsymbol{\beta}$={1,0.1}, randomly allocated gender (*i.e.* X_{i2}=0 or 1 with equal probability along with X_{i1}=1) and one additive genetic effect per individual (*i.e.* $\boldsymbol{Z}=\boldsymbol{I}$).

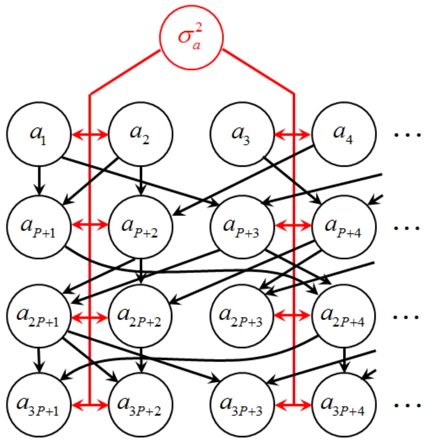

Figure O1: A specific quantitative genetics example in which *a* represent additive genetic effects for a population of *P* individuals randomly mated over four generations (note, for clarity fixed effects β and observations *y* have been omitted from this diagram). For the non-founding population the random effect for each individual has contributions from its two parents in the previous generation.

[7] Individuals are related to themselves through A_{nn}=1 (assuming they are not inbred). If individual *n* is the parent of *p* then A_{np}=½, for siblings sharing the same parents A_{np}=½, for half-sibs A_{np}=¼ and for a grandparent/grandchild relationship A_{np}=¼ *etc...* Whilst $\boldsymbol{A}$ itself is not sparse, its inverse $\boldsymbol{A}^{-1}$ is (only diagonal and parent-sibling elements are non-zero).

Observation model and latent process likelihood

We identify model parameters $\theta = \{\sigma_a^2, \sigma_\varepsilon^2, \boldsymbol{\beta}\}$ and latent variables $\xi = \{\boldsymbol{a}\}$, with residuals $\boldsymbol{\varepsilon}$ incorporated into the observation model.

At first glance it might appear that PBPs are not applicable to this particular model because the latent variables are MVN distributed (*i.e.* a distribution not contained within Table 1). This, however, turns out not to be the case, because of the following transformation.

The latent process likelihood is given by

$$\pi(\xi \mid \theta) = \pi(\boldsymbol{a} \mid \sigma_a^2) = \frac{1}{\sqrt{\left(2\pi\sigma_a^2\right)^E |A|}} e^{-\frac{1}{2\sigma_a^2}\sum_{d=1}^{E}\sum_{e=1}^{E} a_d A_{de}^{-1} a_e}. \tag{O1}$$

Separating out those terms which depend on a_E in the sum (and remembering that $\boldsymbol{A}$ is symmetric and fixed), leads to the product of a normal distribution for a_E (given $a_{e'<E}$) multiplied by a new MVN distribution over the remaining *E-1* latent variables

$$\pi(\boldsymbol{a} \mid \sigma_a^2) \propto f_{\text{norm}}(a_E \mid -\sum_{e'=1}^{E-1} A_{Ee'}^{-1} a_{e'}, \sigma_a^2 / A_{EE}^{-1}) \times \sigma_a^{-(E-1)} e^{-\frac{1}{2\sigma_a^2}\left(\sum_{d=1}^{E-1}\sum_{e=1}^{E-1} a_d \left(A_{de}^{-1} - A_{Ed}^{-1} A_{Ee}^{-1} / A_{EE}^{-1}\right) a_e\right)}, \tag{O2}$$

where the p.d.f. for the univariate normal distribution is given by

$$f_{\text{norm}}(x \mid \mu, \sigma^2) = \frac{1}{\sqrt{2\pi\sigma^2}} e^{-\frac{(x-\mu)^2}{2\sigma^2}}. \tag{O3}$$

The scheme above can be iterated until the original MVN distribution is converted into a product of normal distributions for each of the random effects

$$\pi(\boldsymbol{a} \mid \sigma_a^2) \propto \prod_{e=1}^{E} f_{\text{norm}}(a_e \mid \mu_e^{\text{mod}}, \sigma_e^{\text{mod}\,2}), \tag{O4}$$

where

$$\mu_e^{\text{mod}} = \sum_{e'<e} M_{ee'} a_{e'}, \quad \sigma_e^{\text{mod}\,2} = s_e \sigma_a^2, \tag{O5}$$

and matrix $\boldsymbol{M}$ and vector $\boldsymbol{s}$ are fixed and calculated from $\boldsymbol{A}$ by recursively applying Eq.(O2). Note, Equation (O4) follows the same structure as Eq.(2.1), showing that it represents a DAG (specifically, the one illustrated in Fig. 7(a)).

Under the above transformation, the observation model and latent process likelihood are given by

$$\begin{aligned}
\pi(y \mid \xi, \theta) &= \prod_{i=1}^{N} f_{\text{norm}}(y_i \mid \sum_{f=1}^{F} X_{if}\beta_f + \sum_{e=1}^{E} Z_{ie} a_e, \sigma_\varepsilon^2), \\
\pi(\xi \mid \theta) &= f_{MVN}(\boldsymbol{a} \mid 0, \sigma_a^2 \boldsymbol{A}) \\
&\propto \prod_{e=1}^{E} f_{\text{norm}}(a_e \mid \mu_e^{\text{mod}}, \sigma_e^{\text{mod}\,2}),
\end{aligned} \tag{O6}$$

where i goes over the observations, f goes over the fixed effects and e goes over the random effects.

Importance distributions

Different levels of ID approximation are illustrated in Fig. O2.

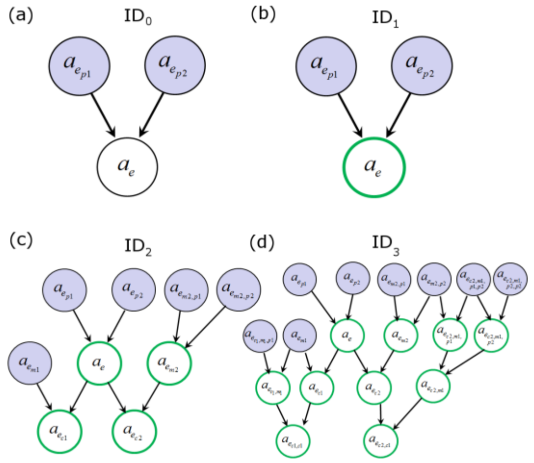

Figure O2: Various levels of approximation used for estimating $f_{ID}(a_e|a_{e'<e},\theta,y)$ for the quantitative genetics model. The shaded circles represent known additive genetic effect $a_{e'<e}$ and the bold, green circles indicate the actual trait measurements used.

ID_0 is given by the model

$$f_{ID_0}(a_e \mid a_{e'<e},\theta) = f_{\text{norm}}\left(a_e \mid \mu_e^{\text{mod}}, \sigma_e^{\text{mod}\,2}\right).$$

From Eq.(4.7), we see that ID_1 is generated by taking the product of the model and the observation probability distributions. For simplicity we assume that each observation contains a single random effect, but that each random effect may have many observations made on it (PBPs can also be applied in the more general case, but estimation of the IDs becomes somewhat more complicated).

A rearrangement of the observation model in Eq.(O4) leads to an effective observation probability for each individual random effect of

$$\pi(y_e \mid a_e,\theta) = f_{\text{norm}}(a_e \mid \mu_e^{\text{obs}}, \sigma_e^{\text{obs}\,2}), \tag{O7}$$

where y_e combines all observations y_i that include random effect a_e, and

$$\mu_e^{\text{obs}} = \frac{\sum_i \left(y_i - \sum_f X_{if}\beta_f\right) Z_{ie}}{\sum_i Z_{ie}^2}, \qquad \sigma_e^{\text{obs}\,2} = \frac{\sigma_\varepsilon^2}{\sum_i Z_{ie}^2}. \tag{O8}$$

Taking the product of the two normal distributions in Eqs.(O4) and (O7) leads to the final result

$$f_{ID_1}(a_e \mid a_{e'<e},\theta,y) = f_{\text{norm}}\left(a_e \mid \frac{\mu_e^{\text{obs}}\sigma_e^{\text{mod}\,2} + \mu_e^{\text{mod}}\sigma_e^{\text{obs}\,2}}{\sigma_e^{\text{obs}\,2} + \sigma_e^{\text{mod}\,2}}, \frac{\sigma_e^{\text{obs}\,2}\sigma_e^{\text{mod}\,2}}{\sigma_e^{\text{obs}\,2} + \sigma_e^{\text{mod}\,2}}\right). \tag{O9}$$

The distribution for ID_2 takes into account those random effects which depend on a_e. As stated in Eq.(4.8) and illustrated in Fig. O2(c), derivation of ID_2 involves integrating over those random effects a_d which depend on a_e. Explicitly writing down the expression for $f_{ID2}(\xi_e|\xi_{e'<e},\theta,y)$ is somewhat verbose. Instead, here we build up the final result by considering different contributions in turn.

To start with we consider a particular random effect a_d (which depends on a_e), and find out how it affects f_{ID2} when it is integrated out. The contribution to the model part of the full posterior from a_d is given by

$$f_{norm}(a_d \mid \textstyle\sum_{e'} M_{de'} a_{e'}, \sigma_d^{\text{mod}\,2}), \tag{O10}$$

where e' sums over all those random effects on which a_d depends. Three possibility exist for e': 1) $e' < e$, in which case $a_{e'}$ is known (as represented by the shaded circles in Fig. O2), 2) $e'=e$ and 3) $e' > e$,

in which case the additional latent variable *e′* has to first be integrated out. In the case of the third option, Eq.(O10) is first recast in terms of the specific variable *e′=r* that needs to be integrated out:

$$f_{norm}(a_r \mid \frac{a_d - \sum_{e'\neq r} M_{de'} a_{e'}}{M_{dr}}, \frac{\sigma_d^{\text{mod}\,2}}{M_{dr}^2}). \tag{O11}$$

Now we remember that the posterior also has a contribution for a_r coming from its measurement and those random effects upon which it depends. These are captured by ID_1 from Eq.(O9), which is given by

$$f_{\text{norm}}(a_r \mid \mu_r^{\text{post}}, \sigma_r^{\text{post}\,2}), \tag{O12}$$

where

$$\mu_r^{\text{post}} = \frac{\mu_r^{\text{obs}}\sigma_r^{\text{mod}\,2} + \mu_r^{\text{mod}}\sigma_r^{\text{obs}\,2}}{\sigma_r^{\text{obs}\,2} + \sigma_r^{\text{mod}\,2}}, \qquad \sigma_r^{\text{post}\,2} = \frac{\sigma_r^{\text{obs}\,2}\sigma_r^{\text{mod}\,2}}{\sigma_r^{\text{obs}\,2} + \sigma_r^{\text{mod}\,2}}. \tag{O13}$$

Multiplying Eqs.(O11) and (O12) integrating over a_r leads to the posterior probability being proportional to

$$e^{-\frac{1}{2\left(\frac{\sigma_d^{\text{mod}\,2}}{M_{dr}^2} + \sigma_r^{\text{post}\,2}\right)}\left(\frac{a_d - \sum_{e'\neq r} M_{de'} a_{e'}}{M_{dr}} - \mu_r^{\text{post}}\right)^2}. \tag{O14}$$

This can again be re-cast in terms of a_d:

$$f_{\text{norm}}(a_d \mid M_{dr}\mu_r^{\text{post}} + \sum_{e'\neq r} M_{de'} a_{e'}, \sigma_d^{\text{mod}\,2} + M_{dr}^2 \sigma_r^{\text{post}\,2}). \tag{O15}$$

Compared to the original expression in Eq.(O10), we see that the effect of integrating out a_r is to replace a_r with the posterior estimate μ_r^{post} in the mean and to add an additional contribution to the variance. The procedure above can be repeated for all *e′>e*, leading to

$$f_{\text{norm}}(a_d \mid \sum_{e'<e} M_{de'} a_{e'} + M_{ee} a_e + \sum_{e'>e} M_{de'} \mu_{e'}^{\text{post}}, \sigma_d^{\text{mod}\,2} + \sum_{e'>e} M_{de'}^2 \sigma_{e'}^{\text{post}\,2}). \tag{O16}$$

We now introduce the contribution which comes from the observation on a_d itself:

$$f_{\text{norm}}(a_d \mid \mu_d^{\text{obs}}, \sigma_d^{\text{obs}\,2}). \tag{O17}$$

Multiplying Eqs.(O16) and (O17), and integrating over a_d implies that the posterior is proportional to

$$e^{-\frac{1}{2\left(\sigma_d^{\text{mod}\,2} + \sigma_d^{\text{obs}\,2} + \sum_{e'>e} M_{de'}^2 \sigma_{e'}^{\text{post}\,2}\right)}\left(\sum_{e'<e} M_{de'} a_{e'} + M_{ee} a_e + \sum_{e'>e} M_{de'} \mu_{e'}^{\text{post}} - \mu_d^{\text{obs}}\right)^2}. \tag{O18}$$

This can be recast in terms of a_e:

$$f_{norm}(a_e \mid \mu_{d\to e}, \sigma_{d\to e}^2), \tag{O19}$$

where

$$\mu_{d\to e} = \frac{\mu_d^{\text{obs}} - \left(\sum_{e'<e} M_{de'} a_{e'} + \sum_{e'>e} M_{de'} \mu_{e'}^{\text{post}}\right)}{M_{ee}},$$
$$\sigma_{d\to e}^2 = \frac{\sigma_d^{\text{mod}\,2} + \sigma_d^{\text{obs}\,2} + \sum_{e'>e} M_{de'}^2 \sigma_{e'}^{\text{post}\,2}}{M_{ee}^2}. \tag{O20}$$

Equation (O19) represents the overall contribution to f_{ID2} from latent variable *d*. To find f_{ID2}, therefore, this distribution must be multiplied over all random effects *d* which depend on a_e, and also the observation and model contributions from a_e itself must be included:

$$f_{\text{norm}}(a_e \mid \mu_e^{\text{post}}, \sigma_e^{\text{post}\,2}) \prod_d f_{norm}(a_e \mid \mu_{d\to e}, \sigma_{d\to e}^2). \tag{O21}$$

Multiplication of these normal distributions yield the final result

$$f_{ID_2}(a_e \mid a_{e'<e}, \theta, y) = f_{\text{norm}}(a_e \mid \mu_e^{ID}, \sigma_e^{ID2}), \tag{O22}$$

where

$$\mu_e^{ID} = \left(\frac{\mu_e^{\text{post}}}{\sigma_e^{\text{post}\,2}} + \sum_d \frac{\mu_{d\to e}}{\sigma_{d\to e}^2}\right)\sigma_e^{ID2},$$
$$\frac{1}{\sigma_e^{ID2}} = \frac{1}{\sigma_e^{\text{post}\,2}} + \sum_d \frac{1}{\sigma_{d\to e}^2}. \tag{O23}$$

Gibbs samplers

Mixed models represent a case for which it is possible to explicitly sample directly from the posterior when model parameters and latent variables are each considered separately [12]. Assuming a simple uniform prior[8], multiplying the two expressions in Eq.(O6) leads to the posterior probability distribution

$$\pi(\sigma_a^2, \sigma_\varepsilon^2, \boldsymbol{\beta}, \boldsymbol{a} \mid \boldsymbol{y}) \propto \frac{1}{\sqrt{(2\pi\sigma_a^2)^E |A|}} e^{-\frac{1}{2\sigma_a^2}\boldsymbol{a}^T \boldsymbol{A}^{-1}\boldsymbol{a}} \prod_{i=1}^N \frac{1}{\sqrt{2\pi\sigma_\varepsilon^2}} e^{-\frac{1}{2\sigma_\varepsilon^2}\left(y_i - \sum_f X_{if}\beta_f - \sum_e Z_{ie} a_e\right)^2}. \tag{O24}$$

The following Gibbs proposals can be identified, which are sequentially applied to constitute a single "update":

Model parameters: Rearranging (O24) gives

$$\pi(\sigma_a^2 \mid \boldsymbol{y}, \sigma_\varepsilon^2, \boldsymbol{\beta}, \boldsymbol{a}) \propto \sigma_a^{-E} e^{-\frac{1}{2\sigma_a^2}\boldsymbol{a}^T \boldsymbol{A}^{-1}\boldsymbol{a}}. \tag{O25}$$

That is, with all other quantities fixed the posterior has an inverted chi-squared distribution with respect to σ_a^2. Similarly, we find that

---

[8] Non-uniform priors can be easily be implemented provided they also take an inverted chi-squared distribution.

$$\pi(\sigma_\varepsilon^2 \mid \boldsymbol{y},\sigma_a^2,\boldsymbol{\beta},\boldsymbol{a}) \propto \sigma_\varepsilon^{-N} e^{-\frac{1}{2\sigma_\varepsilon^2}\sum_{i=1}^{N}\left(y_i-\sum_f X_{if}\beta_f-\sum_e Z_{ie}a_e\right)^2}, \qquad \text{(O26)}$$

and so σ_ε^2 also has an inverted chi-squared distribution. See below for how to draw samples from the inverted chi-squared distributions in Eqs.(O25) and (O26).

Taking each fixed effect β_f in turn, the posterior can be written as

$$\pi(\beta_f \mid \boldsymbol{y},\sigma_a^2,\sigma_\varepsilon^2,\beta_{f'\neq f},\boldsymbol{a}) = f_{\text{norm}}\left(\beta_f \mid \sum_i X_{if}\left[y_i-\sum_{f'\neq f}X_{if'}\beta_{f'}-\sum_e Z_{ie}a_e\right],\frac{\sigma_\varepsilon^2}{\sum_i X_{if}^2}\right). \qquad \text{(O27)}$$

Thus, its value can be sampled from the following normal distribution

$$\beta_f \sim N\left(\sum_i X_{if}\left[y_i-\sum_{f'\neq f}X_{if'}\beta_{f'}-\sum_e Z_{ie}a_e\right],\frac{\sigma_\varepsilon^2}{\sum_i X_{if}^2}\right). \qquad \text{(O28)}$$

Latent variables: Gibbs sampling for random effect a_e is achieved through

$$a_e \sim N\left(\mu_e^{\text{gibbs}},\sigma_e^{\text{gibbs}2}\right), \qquad \text{(O29)}$$

where

$$\mu_e^{\text{gibbs}} = \left(\frac{1}{\sigma_\varepsilon^2}\sum_i Z_{ie}\left[y_i-\sum_f X_{if}\beta_f-\sum_{e'\neq e}Z_{ie'}a_{e'}\right]-\frac{1}{\sigma_a^2}\sum_{e'\neq e}A_{ee'}^{-1}a_{e'}\right),$$
$$\frac{1}{\sigma_e^{\text{gibbs}2}} = \frac{1}{\sigma_a^2}+\frac{\sum_i Z_{ie}^2}{\sigma_\varepsilon^2}. \qquad \text{(O30)}$$

Sampling from the inverse chi-squared distribution

We assume an inverse chi-squared distribution of the form

$$f_\chi(x \mid N,S) \propto x^{-\frac{M}{2}}e^{-\frac{S}{2x}}. \qquad \text{(O31)}$$

A simple method to calculate samples from this distribution is through

$$x=\frac{S}{2\rho},\quad \rho=-\sum_{m=1}^{M-1}\log(u_m), \qquad \text{(O32)}$$

where u_m are uniform randomly generated numbers between 0 and 1.

Appendix P: Details for the logistic population model

In this appendix we provide additional details relevant to the logistic population model in §5.4.

Simulation and prior details

Simulated data was generated using the following parameters: birth rate r_b=0.6, mortality rate μ=0.3, carrying capacity K=100, and capture probability p=0.5. The following priors were used: A gamma distributed prior on μ with mean 0.3 and variance 0.0144, a beta distributed prior on p with

mean 0.5 and variance 0.0025, a uniform prior on *K* between 0 and 200, and a uniform prior on r_b between 0 and 2.

Observation model and latent process likelihood

We identify model parameters $\theta = \{r_b, \mu, K, p\}$ and latent variables $\xi = \{b_t, d_t\}$, which give the number of births and deaths during each time interval *t*.

The observation model and latent process likelihood are given by

$$\pi(y \mid \xi, \theta) = \prod_{m=1}^{M} \frac{P_{m_t}!}{y_m!(P_{m_t} - y_m)!} p^{y_m} (1-p)^{P_{m_t} - y_m},$$
$$\pi(\xi \mid \theta) \propto \prod_{t=1}^{T} \frac{\lambda_t^{b_t}}{b_t!} e^{-\lambda_t} \times \frac{v_t^{d_t}}{d_t!} e^{-v_t}, \qquad \text{(P1)}$$

where *m* goes over all the measurements, y_m are the number of animals observed at time m_t, P_t is the population size, and $\lambda_t = \tau r_b P_t (1 - P_t / K)$ and $v_t = \tau \mu P_t$ are, respectively, the expected number of births and deaths during time interval *t*.

The standard approach

Random walk MH updates are used for the parameters r_b, μ, K, and p (*i.e.* this consists of proposing a new parameter by adding a normally distributed contribution to its existing value and accepting or rejecting that change). The jumping sizes of these separate proposals are individually tuned to give acceptance approximately 33% of the time (using the same procedure as for *j* in appendix C).

Regarding the latent variables, four types of proposal are used: 1) incrementing or decrementing a birth number b_t with randomly selected time *t*, 2) doing the same for a randomly selected death number d_t, 3) scanning from *t*=1 to *t*=*T* and locally incrementing or decrementing both birth number b_t and death number d_t (leaving population sizes unchanged), and 4) scanning from *t*=2 to *t*=*T* and locally incrementing or decrementing the population size P_t (with corresponding adjustments to b_t,d_t and b_{t-1},d_{t-1}). Note these last two options are scanned across all times because here individual proposals are fast (these local changes do not require the entire likelihood and observation model to be calculated).

References

[1] A. Doucet, N. De Freitas, and N. Gordon, "An introduction to sequential Monte Carlo methods," in *Sequential Monte Carlo methods in practice*: Springer, 2001, pp. 3-14.

[2] P. W. Glynn and D. L. Iglehart, "Importance sampling for stochastic simulations," *Management Science,* vol. 35, no. 11, pp. 1367-1392, 1989.

[3] C. Andrieu and J. Thoms, "A tutorial on adaptive MCMC," *Statistics and Computing,* vol. 18, pp. 343-373, 2008/12/01 2008.

[4] G. O. Roberts and J. S. Rosenthal, "Examples of Adaptive MCMC," *Journal of Computational and Graphical Statistics,* vol. 18, pp. 349-367, 2009/01/01 2009.

[5] W. H. Press, *FORTRAN numerical recipes*, 2nd ed. Cambridge England ; New York: Cambridge University Press, 1996.

[6] O. Papaspiliopoulos, G. O. Roberts, and M. Sköld, "A general framework for the parametrization of hierarchical models," *Statistical Science,* pp. 59-73, 2007.

[7] R. M. Neal, "MCMC Using Hamiltonian Dynamics," (in English), *Handbook of Markov Chain Monte Carlo,* pp. 113-162, 2011.

[8] M. Betancourt, "A conceptual introduction to Hamiltonian Monte Carlo," *arXiv preprint arXiv:1701.02434,* 2017.

[9] M. D. Hoffman and A. Gelman, "The No-U-turn sampler: adaptively setting path lengths in Hamiltonian Monte Carlo," *Journal of Machine Learning Research,* vol. 15, no. 1, pp. 1593-1623, 2014.

[10] C. Andrieu, A. Doucet, and R. Holenstein, "Particle markov chain monte carlo methods," *Journal of the Royal Statistical Society: Series B (Statistical Methodology),* vol. 72, no. 3, pp. 269-342, 2010.

[11] C. J. Geyer, "Practical Markov Chain Monte Carlo," (in en), *Statist. Sci.,* vol. 7, no. 4, pp. 473-483, 1992/11 1992.

[12] D. Sorensen and D. Gianola, *Likelihood, Bayesian and MCMC methods in quantitative genetics* (Statistics for biology and health). New York: Springer-Verlag, 2002, p. 740.
